# A comparative study of 3D measuring methods for monitoring breast volume changes

**DOI:** 10.1371/journal.pone.0305059

**Published:** 2024-06-06

**Authors:** Benthe A. M. Dijkman, Niels P. T. J. Liberton, Sjoerd te Slaa, Jan Maerten Smit, Chantal M. Wiepjes, Koen M. A. Dreijerink, Martin den Heijer, Rudolf M. Verdaasdonk, Christel J. M. de Blok

**Affiliations:** 1 Center of Expertise on Gender Dysphoria, Amsterdam UMC, VU University, Amsterdam, the Netherlands; 2 Department of Endocrinology and Metabolism, Amsterdam UMC, VU University, Amsterdam, the Netherlands; 3 Department of Medical Technology, 3D Innovation Lab, Amsterdam UMC, VU University, Amsterdam, the Netherlands; 4 Department of Plastic, Reconstructive and Hand Surgery, Amsterdam Movement Sciences, Amsterdam UMC, VU University, Amsterdam, the Netherlands; 5 Faculty of Science and Technology, Health Technology Implementation, University of Twente, Enschede, the Netherlands; University of Iceland, ICELAND

## Abstract

Three-dimensional (3D) imaging techniques are promising new tools for measuring breast volume, for example in gender-affirming therapy. Transgender individuals can be treated with gender-affirming hormone therapy (GAHT). A robust method for monitoring breast volume changes is critical to be able to study the effects of feminizing GAHT. The primary aim of this study was to compare the accuracy of three 3D devices (Vectra XT, Artec LEO and iPhone XR) for measuring modest breast volume differences using a mannequin. The secondary aim of this study was to evaluate these methods in several performance domains. We used reference prostheses of increasing volumes and compared the volumes using GOM-inspect software. For Vectra XT 3D images, manufacturer-provided software was used to calculate volumes as well. The scanning methods were ranked based on their performance in a total of five categories: volume estimations, costs, user-friendliness, test subject-friendliness and technical aspects. The 3D models analyzed with GOM-inspect showed relative mean estimate differences from the actual volumes of 9.1% for the Vectra XT, 7.3% for the Artec LEO and 14% for the iPhone XR. For the Vectra XT models analyzed with the built-in software this was 6.2%. Root mean squared errors (RMSE) calculated based on the GOM-inspect volume analyses showed mean RMSEs of 2.27, 2.54 and 8.93 for the Vectra XT, Artec LEO and iPhone XR, respectively. The Vectra software had a mean RMSE of 3.00. In the combined performance ranking, the Vectra XT had the most favorable ranking, followed by the Artec LEO and the iPhone XR. The Vectra XT and Artec LEO are the preferred scanners to monitor breast development due to the combination of higher accuracy and overall performance. The current study shows that 3D techniques can be used to adequately measure modest breast volume differences and therefore will be useful to study for example breast changes in transgender individuals using feminizing GAHT. These observations may also be relevant in other fields of 3D imaging research.

## Introduction

The distress caused by the incongruence between a person’s sex assigned at birth and gender identity is termed gender dysphoria [1]. Individuals diagnosed with gender dysphoria can be treated with gender-affirming hormone therapy (GAHT) to induce the desired physical changes and alleviate mental distress [2]. For transgender individuals seeking feminization (in this report, we will use the term trans women; male sex assigned at birth with a female gender identity) GAHT usually consists of anti-androgens and estrogens. GAHT for trans women results in feminization of the body, e.g. breast development, changes in fat distribution and softening of the skin [3–6].

Breast development is an important aspect of feminization and is therefore an important treatment goal of GAHT for most trans women. To date, numerous studies have assessed breast growth in trans women receiving GAHT, using various measurement methods (such as Tanner staging, hemi circumference measurements, tape-measured breast-chest differences and three-dimensional (3D) techniques) [7–10]. 3D imaging represents a promising method for measuring breast development. Studies have shown that 3D imaging techniques are accurate for breast volume assessment. In addition, 3D techniques are non-invasive, affordable and quick [11, 12], in contrast to magnetic resonance imaging (MRI), which is currently considered the gold standard for breast volume assessment [13].

3D models are based on point clouds. There are two major 3D techniques with accompanying devices and 3D software available to create these point clouds: the vertical cavity surface-emitting laser (VCSEL) method creates a 3D model using reflections of laser beams aimed at the surface of the object of interest [14]. Another technique is photogrammetry, which creates a point cloud based on the distance between reference points within multiple photographs [15].

In the transgender research field, monitoring breast development of trans women during GAHT is an emerging area of interest. Breast volume changes in trans women are often modest, increases of less than 100 cubic centimeters (cc) during GAHT treatment are common [10]. It is unknown which 3D device and technique is best-suited to assess breast volume differences in trans women during GAHT. Therefore, this study aimed to find accurate and convenient 3D methods to assess volume changes. For this purpose, we compared the performance of three different 3D imaging devices: the Vectra XT, the Artec LEO and the iPhone XR and performed an assessment of accuracy, costs, user-friendliness, test subject-friendliness and technical aspects, bearing in mind implementation in breast volumetric research in transgender individuals.

## Materials and methods

### Materials

In this study, a Vectra XT scanner, an Artec LEO scanner and an iPhone XR were used.

The Vectra XT (1.2 mm resolution; Canfield Scientific, Parsipanny, NJ, USA) is a photogrammetry device and consists of three pods with six cameras. The Vectra XT has accompanying Vectra software (Vectra Breast Sculptor, v 5.5.7, Canfield Scientific, Parsipanny, NJ, USA). The Artec LEO (0.2mm 3D resolution; Artec 3D, Luxembourg, Luxembourg) is a wireless vertical cavity surface-emitting laser scanner (VCSEL). It can be used with Artec Studio software version 15.1.2.60. The iPhone XR (Apple, Cupertino, CA, USA) is a device with a ‘true-depth’ camera based on the VCSEL-technique. To create a 3D model from the iPhone image, the application Heges (0.5–1.0 mm precision; Heges, North Moravia, Czech Republic) was used. From all systems, a standard triangle language (STL) file can be exported for further analysis.

### Procedures and data collection

**Fig 1:**
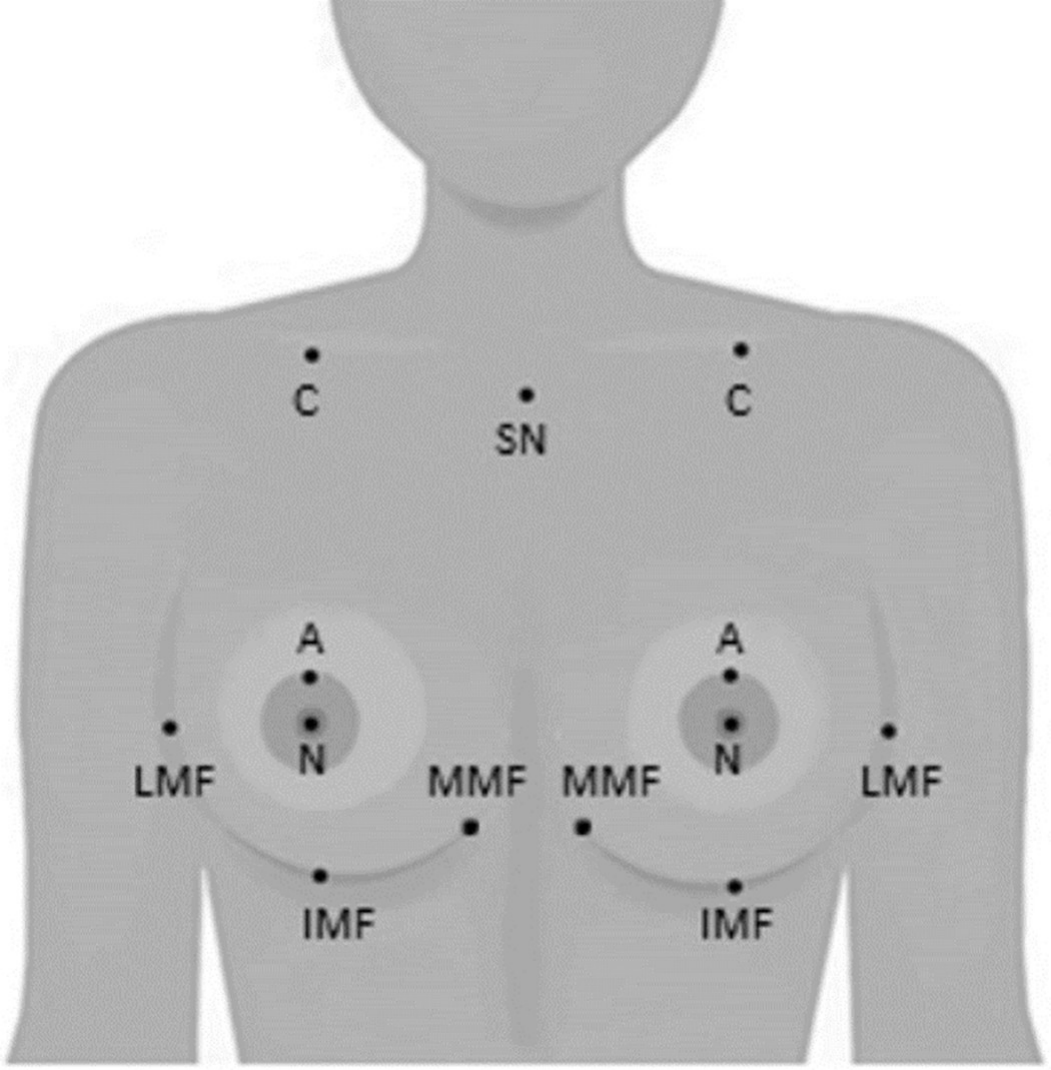
Landmarks on the mannequin. Abbreviations: C = clavicle; SN = sternal notch; N = nipple; A = areola; LMF = lateral mammary fold; IMF = inframammary fold; MMF = medial mammary fold.

A female mannequin with three different reference breast sizes was used as test subject. The different breast sizes were created using clay prostheses of 75 cc, 100 cc and 125 cc. The volume of the clay prostheses was determined using Archimedes’ principle of water displacement [16]. As a control measurement, we used the mannequin without prostheses. To calculate the breast volumes, we performed 20 repeated measurements per volume (i.e. without prostheses, 75 cc add-on, 100 cc add-on or 125 cc add-on) per device. Since the Vectra XT software requires landmarks for breast volume estimations, landmarks were placed on the mannequin model beforehand indicating the sternal notch, both the clavicles, the nipples, the areola borders, the inframammary folds (IMF) and the lateral and medial mammary folds (LMF and MMF) (Fig 1). Since the mannequin did not actually have a sternal notch, clavicles, nipples and areola borders, these landmarks were placed at the approximate location of these landmarks on a real human. The sternal notch and clavicle landmarks were not moved during the measurements. Furthermore, the mannequin had a realistic breast shape. We ensured that the placement of the nipple and areola border landmarks was similar during the different measurements. The IMF was placed at the transition point from the ‘breast tissue’ to the chest wall. To determine the LMF and MMF the natural line and contour of the vertical breast crease of the mannequin was followed. The prostheses were carefully sculpted to match the shape of the mannequin’s breasts, so the prosthesis would fit the mannequin’s chest properly. When a breast prosthesis was placed on the mannequin, the landmarks were modified accordingly when necessary.

### Test set-ups

**Fig 2:**
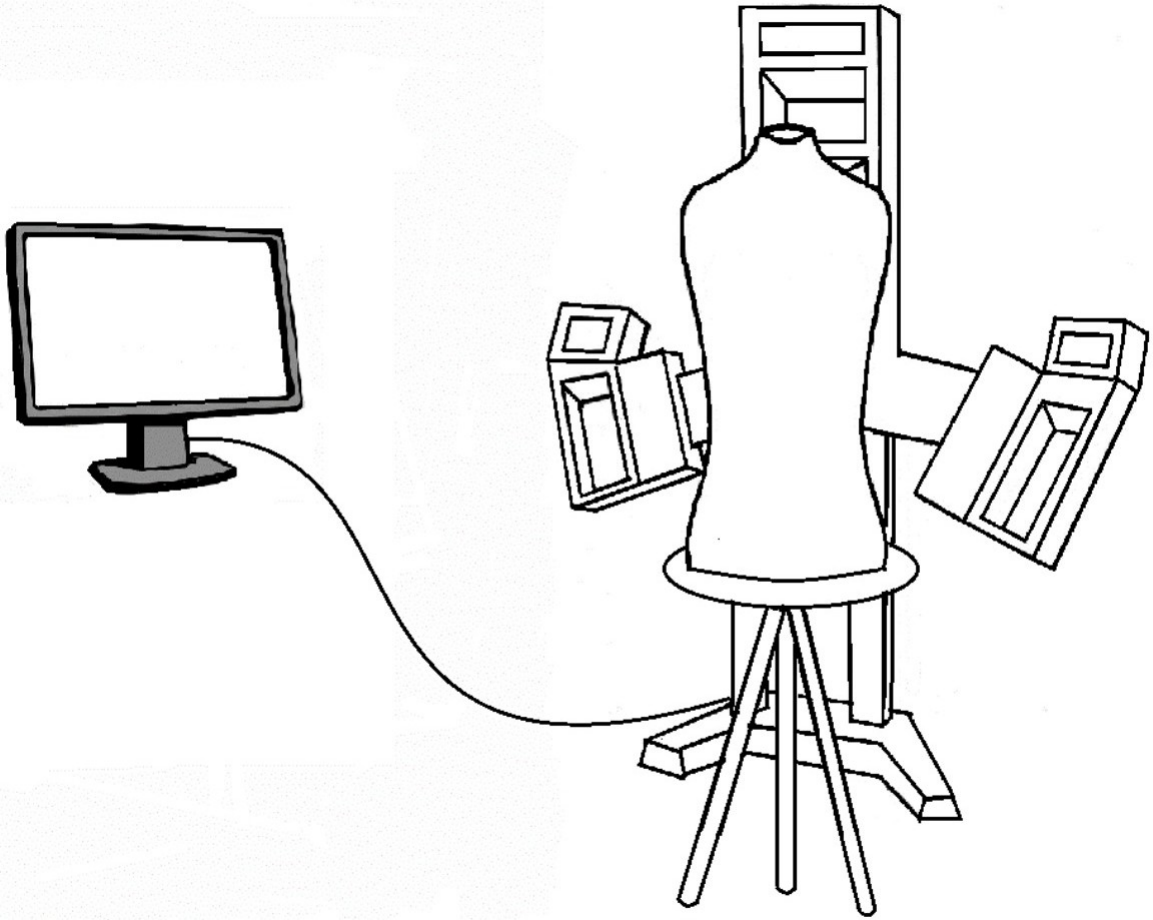
Vectra XT 3D photogrammetry device with three pods, within the pods two cameras and computer.

**Fig 3:**
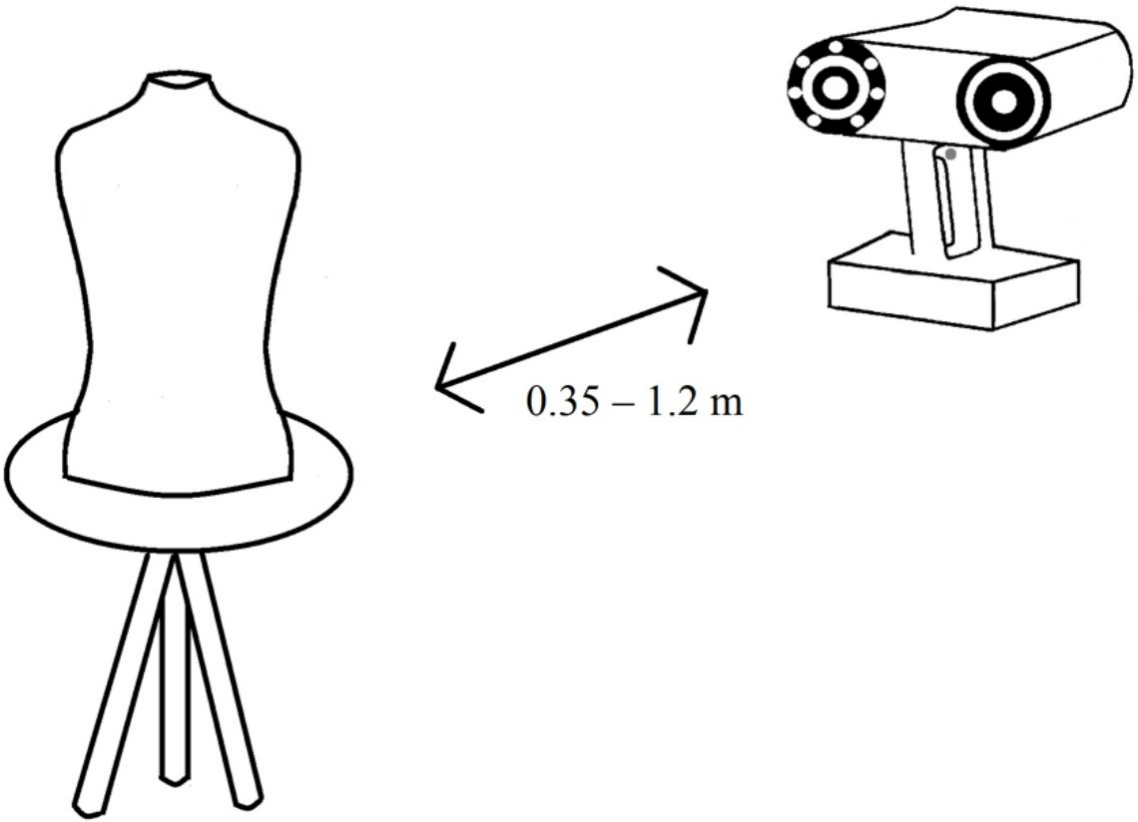
Test set-up with the Artec LEO wireless handheld 3D scanner using VCSEL technology.

**Fig 4:**
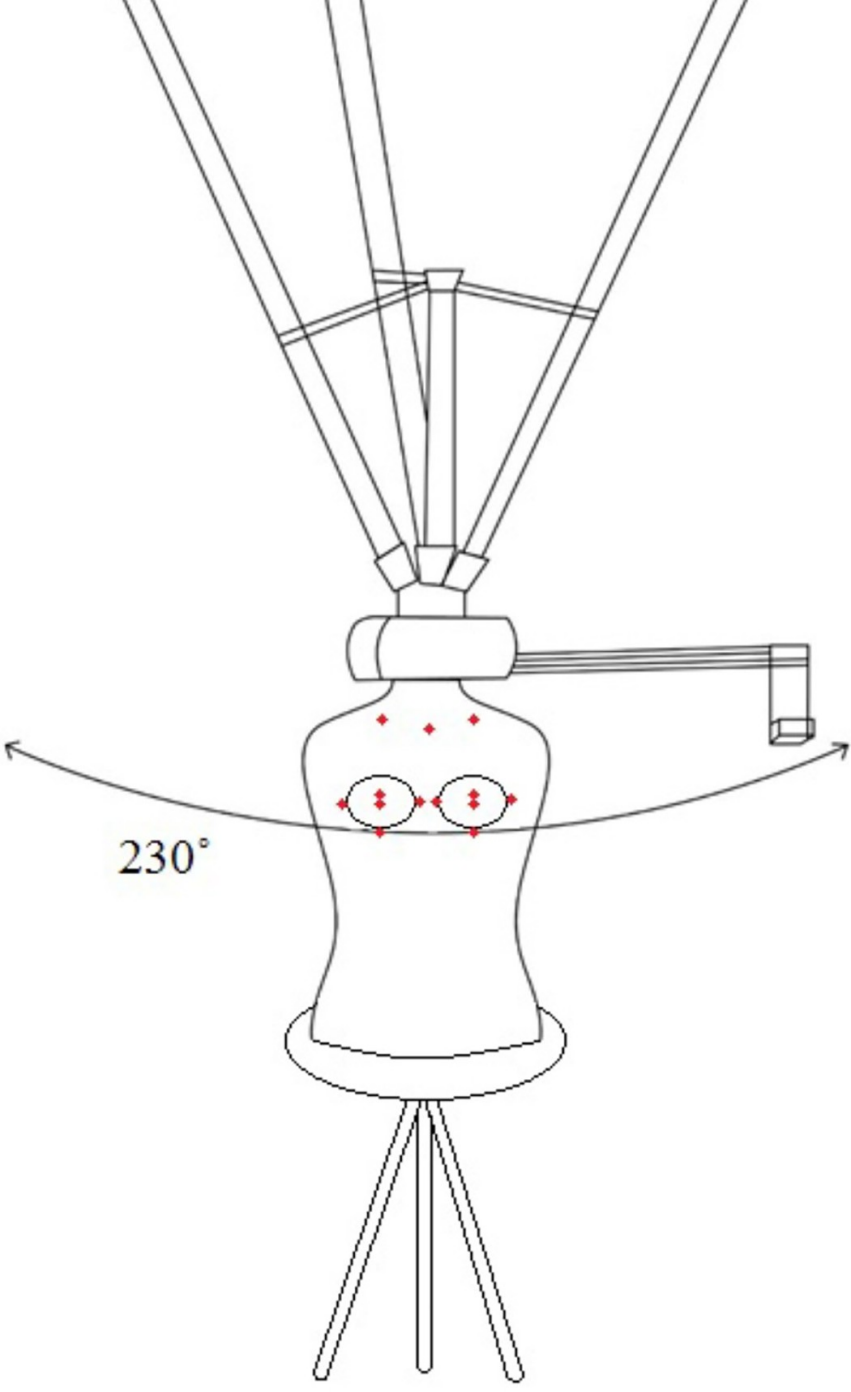
iPhone XR test set-up with the mannequin, tripod and rotating arm with a turning radius of 230°.

The test set-ups were as follows: for the Vectra XT, the mannequin was placed in front of the scanner at a distance of 0.7 meters. The six cameras simultaneously capture a picture. The computer with Vectra Software processes the images into a 3D model (Fig 2). The Artec LEO setup consists of a wireless handheld scanner with two cameras (Fig 3). There is a display with heat map function at the rear side of the device, which serves to track the scanning process. To create a scan, the Artec LEO is moved manually around the mannequin at a distance between 0.35–1.2 meters. Subsequently, Artec software is used to create a 3D model. The iPhone XR set-up consisted of a tripod hanging from a ceiling (Fig 4). A remotely controlled rotating arm was attached to the tripod with an additional phone holder attached to the end of the arm. The rotating arm was controlled through the corresponding application Syrp on the researcher’s smartphone (Syrplab, Auckland, New Zealand). The maximum rotation of the arm was 230°. The application Heges was used to scan the mannequin and create the 3D model.

All scanning methods were assessed on their overall performance in five categories: volume estimations (described above), costs, user-friendliness, test subject-friendliness and technical aspects. In each category, a method was given either 3 points (high), 2 points (moderate), or 1 point (low). The prices of the devices were retrieved from the companies’ websites in 2021. The following aspects were considered: user friendliness of the scanner including the scanning time, test-subject friendliness of the scanner, time and effort to transfer files for analysis, time for the software take to create a 3D model. During the analyses with the GOM-inspect software and the Vectra XT built-in software we focused on the quality of the 3D models, the accuracy of the breast volume measurements compared to the reference values and the technical aspects of the analysis software.

### Statistical analysis

All 3D scan files were analyzed in GOM-inspect for breast volume estimation (GOM, Braunschweig, Germany). We also included the Vectra built-in software for comparison. The GOM-inspect software estimates breast volume based on aligned, cut and filled models. Borders of the region of interest were similar for each 3D model. The Vectra XT software estimates breast volumes based on a region of interest indicated by landmarks. The landmarks in the software were matched manually with the landmarks on the mannequin.

The mean baseline volume was then subtracted from the 75 cc, 100 cc and 125 cc volume estimations in order to calculate the volume difference. Linear regression was used to estimate the mean differences from the expected volume. Subsequently, we calculated the relative differences in estimate from the volume of each prosthetic breast in percentages. Root mean squared errors (RMSE) were calculated to estimate the accuracy of the volume estimations by each individual 3D device. RMSE is a measure of the difference between the predicted value and the observed value [17]. A lower RMSE indicates a higher accuracy. To assess the quality of the 3D models, surface geometries of the baseline models of each device were compared with surface deviation analysis. Surface deviations were calculated as arithmetic means in millimeters (mm). The statistical analyses in this study were executed in the statistical analysis program STATA (Statistical Software: Release 15. StataCorp LLC, College Station, TX, USA).

## Results

### Volume estimations

The results of the volume estimations are shown in Table 1 and all volume measurements are included in S1 Table. The Vectra XT models analyzed with GOM-inspect showed a pooled mean volume difference of 8.1 cc and a relative mean difference of 9.1%. For the Artec LEO the pooled mean volume difference was 6.8 cc. The relative mean real difference for the Artec LEO was 7.3%. Lastly, the iPhone XR showed a pooled mean volume difference 15.1 cc, with a relative mean difference of 13.5%. For the Vectra XT models, analyzed with the built-in software, results showed a pooled mean volume difference of 5.8 cc and a relative mean difference of 6.2%.

**Table 1:** Volume differences from expected volume per scanner.

3D technique	75 cc Right	Left	100 cc Right	Left	125 cc Right	Left	Pooled Mean (SD)
**Vectra XT (GOM)**	13.0 [12.0–14.0] 17.3%	14.8 [13.7–15.9] 19.7%	-1.8 [-2.9 –-0.7] 1.8%	-3.5 [-4.4 –-2.6] 3.5%	10.1 [8.6–11.5] 8.1%	5.4 [4.6–6.3] 4.1%	8.1 (5.3) 9.1% (7.6)
**Artec LEO (GOM)**	7.1 [5.9–8.3] 9.5%	9.0 [6.8–11.2] 12.0%	-7.8 [-8.6 –-7.0] 7.8%	-6.4 [-7.4 –-5.5] 6.4%	-4.0 [-5.1 –-3.0] 3.2%	6.3 [5.4–7.3] 5.0%	6.8 (1.7) 7.3% (3.2)
**iPhone XR (GOM)**	23.8 [19.0–28.6] 31.7%	11.0 [6.3–15.6] 14.7%	4.7 [0.6–8.8] 4.7%	9.3 [4.3–14.3] 9.3%	20.3 [17.0–23.6] 16.2%	21.5 [18.3–24.7] 4.3%	15.1 (7.8) 13.5% (10.2)
**Vectra XT Software**	11.9 [10.2–13.7] 15.9%	-0.04 [-1.8–1.7] 0.1%	-3.8 [-5.3 –-2.2] 3.8%	-9.4 [-10.5 –-8.4] 9.4%	9.1 [7.8–10.3] 7.3%	0.7 [-0.4–1.8] 0.6%	5.8 (5.0) 6.2% (6.0)

*Note*. cc = cubic centimeters. Numbers between brackets represent 95% confidence intervals. Pooled Mean represents the mean of all volume differences per device. Negative difference scores were rescored into positive differences for the calculation. Percentages indicate the relative difference in estimate from the real value.

### Accuracy

The RMSE results are shown in Table 2. RMSEs calculated based on the GOM-inspect volume analyses showed a mean RMSE of 2.27 for the Vectra XT. For the Artec LEO, RMSEs calculated based on the GOM-inspect volume analyses showed a mean RMSE of 2.54. RMSEs calculated based on the GOM-inspect volume analyses of the iPhone XR showed a mean RMSE of 8.93. RMSEs calculated based on the Vectra’s built-in software volume analyses showed a mean RMSE of 3.00.

**Table 2:** Root-mean-squared errors (RMSE) per scanner.

3D technique	75 cc Right	Left	100 cc Right	Left	125 cc Right	Left	Pooled Mean (SD)
**Vectra XT (GOM)**	2.15	2.44	2.27	1.92	3.12	1.71	2.27 (0.49)
**Artec LEO (GOM)**	2.62	4.72	1.77	1.93	2.21	2.01	2.54 (1.11)
**iPhone XR (GOM)**	10.3	9.99	8.86	10.6	6.95	6.86	8.93 (1.67)
**Vectra XT Software**	3.78	3.66	3.31	2.18	2.67	2.40	3.00 (0.68)

*Note*. RMSE scores were calculated by the difference between the gold standard and estimated volumes.

### Comparative analysis

**Fig 5:**
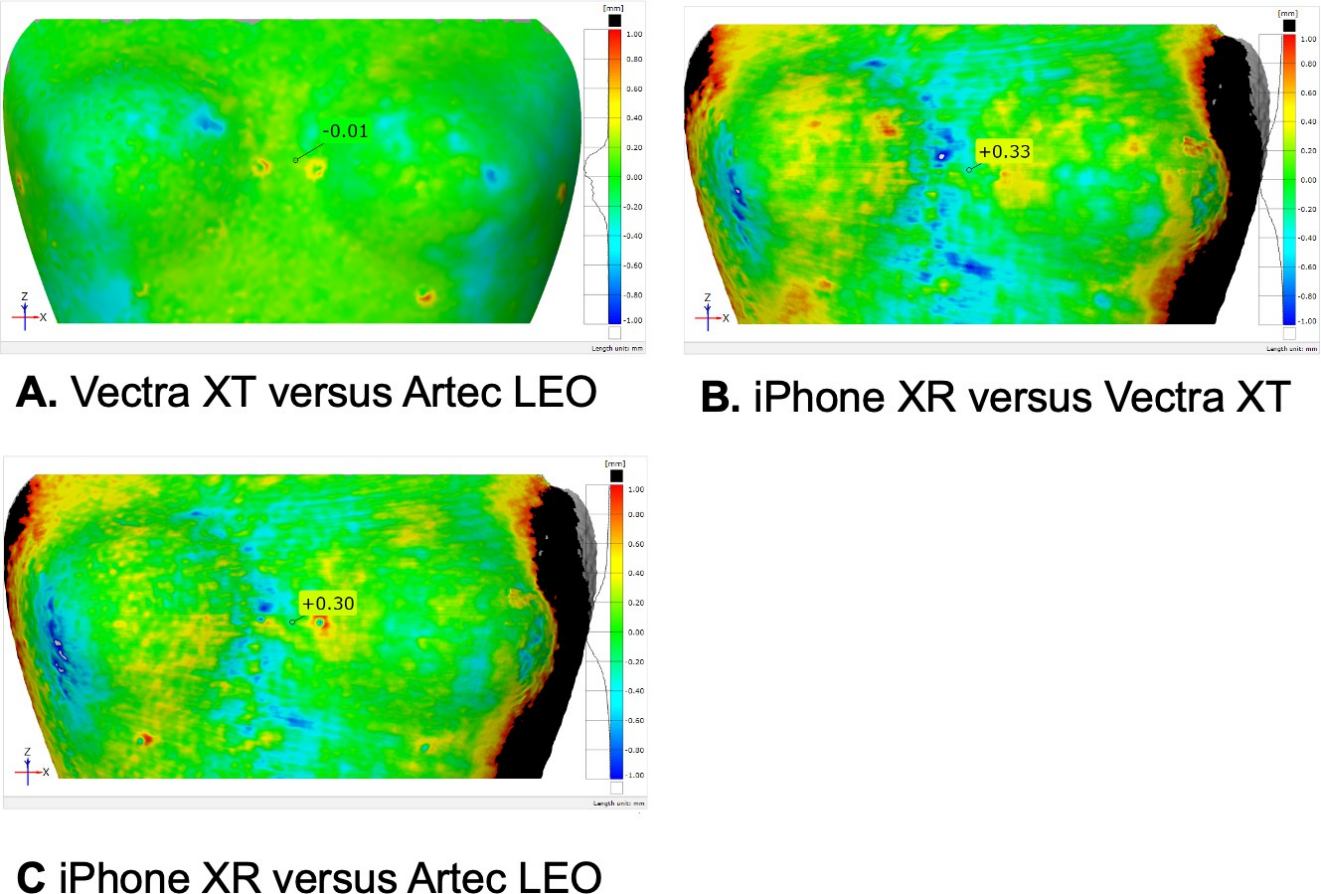
GOM-inspect 3D deviation analyses of the baseline models. (A) Vectra XT versus Artec LEO, (B) iPhone XR versus Vectra XT, (C) iPhone XR versus Artec LEO. Surface deviations are indicated by the coloured scale on the right. Red indicates a positive surface deviation, blue indicates a negative surface deviation. Arithmetic means are shown in the middle: A -0.01 mm, B +0.33 mm, C +0.30 mm.

The surface deviation analyses in GOM-inspect based on the 3D models without prostheses, showed high correlation between the Vectra XT and Artec LEO 3D methods (arithmetic mean = -0.01 millimeters (mm)) (Fig 5A). The iPhone XR compared to the Vectra XT showed an arithmetic mean of +0.33 mm (Fig 5B). The iPhone XR compared to the Artec LEO showed an arithmetic mean of +0.30 mm (Fig 5C).

### Performance ranking

A complete summary of the costs, user-friendliness, test subject-friendliness and technical aspects is shown in Table 3. In total, the Vectra XT had 13 points, the Artec LEO 12 points and the iPhone XR 9 points (Table 4).

**Table 3:** Summary of the costs, user-friendliness, test subject-friendliness and technical aspects of each 3D method.

	Costs	User-friendliness	Test subject-friendliness	Technical aspects
**Vectra XT**	€33,500	+ Straight forward procedure for capturing 3D image —Large room is needed	++ Speed: 3.5 milliseconds ++ Non-invasive	+ Built-in software which can estimate absolute cc’s per breast: 5 minutes + Can be easily analyzed in GOM-inspect: 10 minutes + High quality 3D models - Different placing of landmarks in built-in software changed the estimated volumes
**Artec LEO**	€23,500	+ Straight forward procedure for scanning ++ Portable	+ Speed: 10 seconds ++ Non-invasive	+ Can be easily analyzed in GOM-inspect: 10 minutes + High quality 3D models - No dedicated volume estimation software
**iPhone XR**	€1,034	- Large space is needed	+ Speed: 10 seconds ++ Non-invasive	+ Can be easily analyzed in GOM-inspect: 10 minutes. - Lower quality 3D models - No dedicated volume estimation software

*Note*. Functionalities are rated on a 5-point scale ranging from—(very bad) to ++ (very good)

**Table 4:** Scores on volume estimations, costs, user-friendliness, test subject-friendliness and technical aspects.

	Volume estimations	Costs	User-friendliness	Test subject-friendliness	Technical aspects	Total
**Vectra XT** *	3	1	3	3	3	13
**Artec LEO**	2	2	2	2	3	11
**iPhone XR**	1	3	1	1	1	7

*score is based on the Vectra XT volume estimations with built-in software

The devices were given 3 points (high), 2 points (moderate) or 1 point (low) per category.

## Discussion

This study focused on finding accurate 3D imaging techniques to estimate breast volume changes. A comparison of three devices for breast volume measurement was conducted and we found that all 3D techniques accurately estimate breast volume, in line with previous studies [12, 18]. Moreover, we found that these methods can be used to measure modest breast volume changes, which is clinically relevant for transgender individuals undergoing feminizing GAHT often resulting in small breast volume changes [7]. The photogrammetry technique used in the Vectra XT, analyzed with the built-in software, was the most accurate to estimate breast volume differences. The volume measurements of the Artec LEO (using VCSEL) and Vectra XT analyzed with GOM, were comparable and only small differences compared to the Vectra XT built-in software were found. The RMSE results revealed that the Vectra XT exhibited the highest accuracy. The Artec LEO followed closely, indicating slightly less accuracy compared to the Vectra XT. The iPhone XR, on the other hand, demonstrated the lowest accuracy, suggesting arguably more deviation from actual breast volumes. These findings collectively indicate that Vectra XT emerges as the most accurate device for breast volume measurement, followed by the Artec LEO, while the iPhone XR falls more behind in its accuracy. This study also showed that the Vectra XT and Artec LEO scanners did not consequently over- or underestimate volumes. Furthermore, surface deviation analyses were performed to compare the surface geometries of the three devices. The low arithmetic mean of the deviation between the Vectra XT and Artec LEO indicated that the surfaces of the Vectra XT and Artec LEO baseline models were most similar. The arithmetic mean of the deviation between the Vectra XT and iPhone XR suggested that the baseline model of the iPhone XR is slightly larger than the baseline model of the Vectra XT. The arithmetic mean of the deviation between the Artec LEO and iPhone XR further supports this. This could explain the volume overestimations in the iPhone XR models.

A recent study compared 3D imaging using a Vectra XT system with MRI with a 3D method and found that 3D imaging with was reliable and showed a high agreement between two measurements [19]. Accordingly, our study showed consistent measurements of the Vectra XT based on the found RMSEs. Our findings confirm and expand a recent preliminary report on the use of the Vectra scanner in transgender breast surgery planning [20]. In addition, our study showed that the iPhone XR is a relatively low cost and fairly accurate 3D method which is supported by previous research [21].

The overall performance analysis showed that the Vectra XT and Artec LEO did well with regard to subject-friendliness, user-friendliness and technical aspects. The iPhone XR is the most cost-efficient option. Although our methods for assessing the performance of the scanners are arbitrary, we found that in general 3D techniques are user- and test-subject friendly, which is in line with to previous research that argued that 3D techniques are more practical, less expensive and less invasive for a test-subject than MRI [12]. 3D techniques have the potential to contribute to routine breast volume measurements in clinical care due to the simplicity and precision of these techniques [11].

The strength of this study is the robust study design using the mannequin with pre-defined reference breast sizes eliminating potential biological variation. Hence, the limitation is the lack of real-life diversity in this test set-up.

In addition to GAHT effects, the results from this study may be useful for the implementation of 3D scanning in the pre- and post-operative breast surgery setting, e.g. for assessing symmetry in breast reconstructive surgery. Beyond breast development and surgery, our observations could also be relevant in other fields of 3D imaging research, in medicine but also very broadly in non-medical applications of 3D scanning and printing. Future validation studies should therefore be carried out in actual human subjects.

From the results of this study, we conclude that 3D techniques can be used to adequately measure modest breast volume differences and could eventually improve research with regard to breast volume changes in transgender individuals.

## Supporting information

S1 Table Volume measurement results. Measurements in cc using scanner types Vectra + GOM (1), Artec LEO + GOM (2), Iphone + GOM (3), Vectra built-in software (4).
(XLSX)
